# Methodological choices in size and density fractionation of soil carbon reserves – A case study on wood fiber sludge amended soils

**DOI:** 10.1016/j.heliyon.2024.e24450

**Published:** 2024-01-10

**Authors:** Riikka Keskinen, Johanna Nikama, Joel Kostensalo, Mari Räty, Kimmo Rasa, Helena Soinne

**Affiliations:** a Natural Resources Institute Finland (LUKE), Tietotie 4, FI-31600, Jokioinen, Finland; b Natural Resources Institute Finland (LUKE), Yliopistokatu 6 B, FI-80100, Joensuu, Finland; c Natural Resources Institute Finland (LUKE), Halolantie 31 A, FI-71750, Maaninka Finland; d Natural Resources Institute Finland (LUKE), Latokartanonkaari 9, FI-00790 Helsinki, Finland

**Keywords:** Organic amendments, Soil conditioning, Carbon storage, Method development

## Abstract

Soil organic carbon (SOC) is in the focus of research due to its central role in regulating climate and maintaining fertility and resilience of soils. Methodologically, shifting from whole soil C measurements to specific SOC fractions increases possibility to detect small changes in the vast SOC storage, and enhances estimation of SOC stability. However, SOC fractionation schemes are numerous and variable. In this study, deionized water and sodium hexametaphosphate (SHMP) were compared in soil dispersion by separating soils into coarse (0.25–2 mm), medium (0.063–0.25 mm) and fine (<0.063 mm) size fractions. The first two fractions were further separated by density (1.8 g cm^−3^) to obtain free particulate organic C (POC) and mineral associated organic C (MOC). The approach was applied to a clay and a silt loam soil with and without wood fiber sludge amendment to follow the added C. Aggregate disruption was enhanced with SHMP in comparison to water, but the effect was small and the use of SHMP decreased recovery of SOC, wherefore water was preferred. In both soils, 5–10 % of SOC occurred as coarse POC, 1–3% as coarse MOC, 5 % as medium POC, 10 % as medium MOC, and 70–85 % as fine MOC. The added C resided in the POC fractions with an indication of minor accumulation to the fine MOC in the clay soil. Longer time frame with repeated C additions would be needed to increase the stable MOC storages though saturation of the MOC reserve may hinder accumulation in the silt loam low in fines.

## Highlights

• In comparison to whole soil carbon analysis fractionation gives more detailed data.
• Sodium hexametaphosphate promotes dispersion but decreases carbon recovery.
• Density separation is needed due to coarse sized mineral associated carbon.
• Roughly half of the applied carbon was recovered 0.5–1 year after application.
• Organic amendment increased free particulate fraction vulnerable to degradation.

## 1 Introduction

Soil organic carbon (SOC) has been brought to the spotlight due to its twofold significance for society. As a major C stock, SOC plays an important role in global climate regulation [[1], [2]]. At the same time, soil organic matter (SOM) is an essential component of soil fertility and resilience, including nutrient cycling, water availability, and biodiversity, thus forming the basis for sustainable agronomic production and food security [3,4]. Consequently, conserving existing as well as restoring depleted SOC stocks is of utmost importance [[5], [6], [7]]. Sustainable SOC management requires knowledge of SOC stabilization mechanisms and means to reliably assess the efficacy of various C accumulating measures in different environments. Observing changes in the SOC pool by direct analyses is challenging due to the small magnitude of the change relative to the absolute concentration and its spatial variability [8,9]. Methods targeting specific SOC fractions, instead of the total pool, are needed in order to have sufficient sensitivity to detect the occurring changes, and to shed light on the processes behind the SOC dynamics [10].

Currently, no consensus exists on the methodology of SOC fractionation, but various schemes based on chemical or physical separation, or their combination, are being used [11]. Chemical methods in general aim to segregate C pools of varying lability or recalcitrance through solubilization, oxidation, or hydrolysis of organic matter with different reagents, whereas physical methods rely on size and/or density separation as the basis of SOC stabilization via interaction with the soil mineral phase [[12], [13], [14]]. Freely occurring particulate organic matter (POM) is vulnerable to microbial attack unless occluded within aggregate structures, whereas mineral associated organic matter (MOM) is protected against decomposition [15,16]. Though the main principles of separating the POM and MOM fractions are consistent, there is much variation in the details, e.g., in means of dispersion and density limits used. Case specific choices and adjustments in the methodology may be needed [17]. Furthermore, terminology is used variably, especially regarding POM, which may refer to coarser size fractions (including both free light material and mineral associated heavy material) or distinctly to density separated light fractions [14,18].

Soils have been amended with organic inputs since ancient times to improve crop productivity through the supply of nutrients, pH regulation, improved structure, and increased nutrient and water holding capacities [19,20]. Recently, increasing the stable C pool of soils has become a secondary goal in addition to maintaining soil fertility when applying various soil conditioners [21]. Among organic side streams, wood fiber sludges of the pulp and paper industry comprise an organic C source with potential to be used for soil amending instead of incineration for energy [[22], [23], [24]]. The composition of these sludges varies according to the source and process conditions, but a considerable proportion of their biomass has been found to be recalcitrant against degradation [[25], [26], [27]]. Therefore, long-term effects following land application may be expected.

In this study, samples from unamended and wood fiber sludge amended clay and silt loam soils were size and density fractionated with the aim of fine-tuning the fractionation methodology by comparing the efficiency of soil dispersion in water to that in sodium hexametaphosphate. In addition, density separation and selection of density limits used are discussed. Furthermore, the effect of added C on the C fractions of the two deviating soils is assessed. The results serve in method selection of future studies and further our understanding on the stability of wood-fiber-sludge-derived C in soil.

## 2 Materials and methods

### 2.1 Studied soils and soil sampling

Soil samples were collected in May 2021 from two field experiments exploring the agronomic and environmental effects of wood fiber sludge amendments. In Jokioinen, south-western Finland, three types of fiber products had been applied with five replicates in a randomized complete block design on a clay textured soil (Luvic Stagnosol) first in September 2015 [24] and then again in October 2020. For this study, soils from unamended control plots and plots having received lime-stabilized pulp mill sludge (LPMS in Ref. [24], N fertilization rate 80 kg ha^−1^) were used. The other experimental site sampled was located in Maaninka, east-central Finland, on a silt loam (Dystric Arenosol) and contained plots in a randomized complete block design with additions of two types of fiber materials (side streams from pulp and paper industry) combined with both a typical and a reduced mineral nitrogen (N) fertilization level each in four replicates. From this experiment established in June 2020, unamended control treatment and unstabilized and non-productized sludge treatment with typical N supply (80 kg N ha^−1^) were sampled for this study. Selected properties of the soils and added fiber materials analyzed previously within the two field studies are presented in Table 1.

**Table 1:** Selected properties of the soils and wood fiber sludge amendments included in the study.

	Study site	
	Jokioinen Clay (Luvic Stagnosol)	Maaninka Silt loam (Dystric Arenosol)
Soil properties		
Clay (<0.002 mm), %	47	13
Silt (0.002–0.063 mm), %	41	50
Fine sand (0.063–0.2 mm), %	6	30
Coarse sand (0.2–2 mm), %	6	7
pH (H_2_O)	6.4	6.0
Total C, %	2.3	1.8
Total N, %	0.19	0.13
Fiber properties		
Total C, %	34.8	34.8
Total N, g kg^−1^	9.8	15.0
Added C, Mg ha^−1^	9.0^a^	3.2

a The amount of C added in 2020, in addition 9.0 Mg C ha^−1^ was applied in September 2015.

Plots from four blocks were sampled from both sites with an auger of ca. 2 cm in diameter such that one composite sample per plot (6 × 15 m in Jokioinen and 1.5 × 15 m in Maaninka) was aggregated from 10 to 15 subsamples collected across the plot from the surface layer (0–10 cm) corresponding to the mixing depth of the fiber amendments. The total number of samples was thus 8 per site. The soils were stored in their initial moisture state at +4 °C until the analyses in July 2021. Drying of the samples was avoided to prevent strengthening of soil aggregates [28] as fractionation by particle size instead of aggregate size was sought [11].

### 2.2 Particle size and density fractionation of SOM

**Fig. 1:**
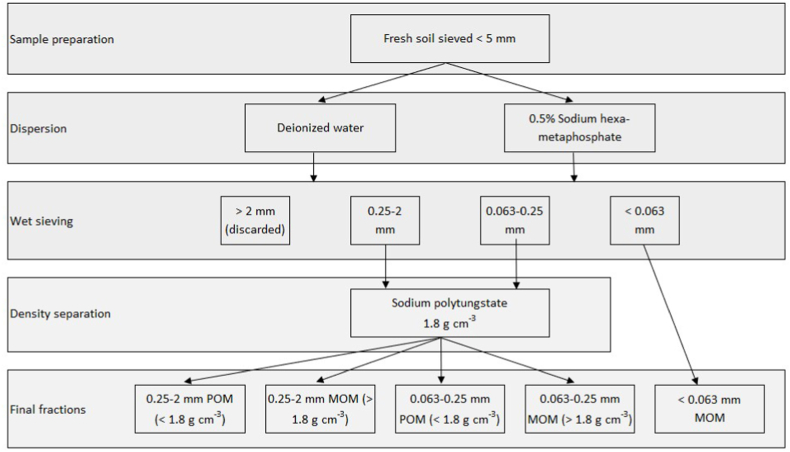
Schematic presentation of the fractionation scheme applied in the study.

Fresh soil samples were first passed through a 5-mm sieve while manually removing visible stones, coarse roots, and crop residues. For separating size and density fractions of SOM (Fig. 1), 50 g of sieved soil was weighed into a 500-ml plastic bottle. The soil was dispersed by adding 15 glass beads (ø 4 mm) and 400 ml of either deionized water or 0.5 % Sodium hexametaphosphate (SHMP) and shaking the suspensions in an orbital shaker for 18 h at 105 rpm seeking for full dispersion. Thereafter, the soils were thoroughly wet sieved with deionized water on sieves of 2 mm, 0.25 mm, and 0.063 mm mesh in succession. The size classes used follow the boundaries of texture classes such that the finer cut-off separates silt and sand, while the coarser cut-off separates fine and coarse sand. Material remaining on top of the 2-mm sieve in addition to the glass beads (single stones and coarse plant residues) was discarded. Particles in size ranges of 0.25–2 mm and 0.063–0.25 mm on top of the 0.25 mm and 0.063 mm sieves, respectively, were carefully transferred into aluminum tins and oven dried to constant weight at 80 °C. The rinsed soil suspension (approx. 3 l) that had passed through all the sieves and contained solids of <0.063 mm and dissolved material was pre-evaporated at 40 °C in plastic buckets and then transferred into aluminum tins and dried at 80 °C.

The dry weight of isolated size fractions was recorded, and the soil material was quantitatively transferred into 50-ml plastic test tubes. Thereafter, the samples of 0.25–2 mm and 0.063–0.25 mm size fractions were further density separated in sodium polytungstate (SPT) adjusted to 1.8 g cm^−3^ density [29]. In preliminary tests, a density of 1.6 g cm^−3^ was used but with this cut-off limit the separation between low and high density fractions remained vague as no distance of clear solution formed between the fractions. The density separation was carried out in the test tubes, in which 25 ml of SPT solution was added and the suspension shaken in an orbital shaker for 30 min followed by a 30-min centrifugation at 2000×*g*. The floating fraction was aspirated on a 12 μm polycarbonate membrane filter with a gentle vacuum and rinsed with deionized water for removing the SPT. The heavy fraction remaining in the bottom of the tube was washed onto a 0.063 mm sieve and thoroughly rinsed with deionized water. Thereafter, the fractions were separately transferred on Petri dishes, dried at 80 °C and weighed.

Finally, the total C concentration of all samples was analyzed via dry combustion (Leco 628 CHN Determinator). The total C in the initial undiluted SPT solution and used SPT solution passed through the 12 μm membrane were also measured and found to be below the detection limit of the equipment (<800 μg C g^−1^ SPT). In addition, the moisture content of the initial fresh soil was determined gravimetrically at 80 °C and thereafter, its total C contents were analyzed similarly than for the separated fractions to allow calculation of mass and C recoveries. In comparison of mass recoveries, the weights of the finest fractions were corrected for the amount of SHMP added.

### 2.3 Statistical methods

The role of dispersion methods on the fractionation efficiency was studied using a linear mixed model. The response variables were first log-transformed in order to obtain roughly normally distributed residuals. For modeling the effect of dispersion on response y the $j^{th}$ observation in block i, i.e., $y_{ij}$ is given by

$$\log\;\left(y_{ij}\right)=\beta_{0}+\beta_{2}Treatment+\beta_{3}Dispersion+b_{i}+\epsilon_{ij}$$

$$b_{i}∼N\left(0,\sigma_{loc}^{2}\right)$$

where Treatment is 0 for the control and 1 for FS, Dispersion is 0 for water dispersion and 1 for hexametaphosphate, $b_{i}$ is a normally distributed block-specific random effect, $\beta_{0,1,2,3}$ are the fixed effects, and $\epsilon_{ij}$ is a normally distributed error term. The variance of the block-specific random effect is free to differ between the locations. For studying the final fractionation, only deionized water was considered simplifying the model to

$$\log\;\left(y_{ij}\right)=\beta_{0}+\beta_{1}Location+\beta_{2}Treatment+b_{i}+\epsilon_{ij}$$

$$b_{i}∼N\left(0,\sigma_{loc}^{2}\right)\text{.}$$

The fitting and relevant inferences were carried out using the R-package nlme [30]. The pairwise differences between treatments were calculated as contrasts from the linear mixed-effects model. All statistical analyses were carried out using the statistical software R [31].

## 3 Results and discussion

### 3.1 Dispersion efficiency

**Fig. 2:**
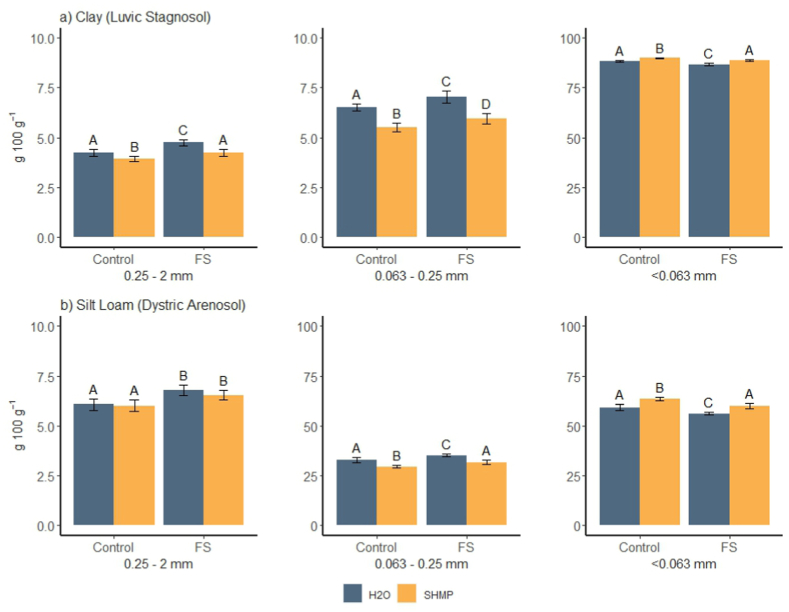
Masses (g 100 g^−1^ soil) of 0.25–2 mm, 0.063–0.25 mm and <0.063 mm size fractions in untreated (Control) and wood fiber sludge -amended (FS) a) Clay (Luvic Stagnosol) and b) Silt Loam (Dystric Arenosol) soils recovered after dispersion in deionized water (H_2_O) or sodium hexametaphosphate (SHMP). Note the different scales on the y-axes. The values are averages of 8 replicates ± standard error. Significant differences between treatments within soil types are denoted with different letters (p < 0.05). The pairwise p-values have been computed based on contrasts from the linear mixed-effects model.

The overall mass recovery of soil in the size fractions was high, 99.2 ± 0.1 % (mean ± SEM). The textural difference between the two sites was clearly reflected in the results such that in the silt loam soil of Maaninka, the masses of the two coarser fractions, especially the 0.063–0.25 mm fraction, were larger and the mass of the fine (<0.063 mm) fraction was smaller than in the clay soil of Jokioinen (Fig. 2).

Sodium hexametaphosphate enhanced soil dispersion in comparison to deionized water since less soil remained in the coarse and medium size fractions (0.25–2 mm and 0.063–0.25 mm) of the clay and in the medium size fraction of the silt loam with SHMP as the dispersing agent (Table 2, Fig. 2). Furthermore, more soil ended up in the fine fraction (<0.063 mm) in both soils when dispersed with SHMP instead of water. In the clay soil, the relative SHMP-induced mass decrease was approximately 10 % in the 0.25–2 mm fraction and 15 % in the 0.063–0.25 mm fraction, while the <0.063 mm fraction increased by ca. 2 %. In the silt loam soil, the decrease was 10 % in the 0.063–0.25 mm fraction and the mass of <0.063 mm fraction increased by 7 %. This indicates SHMP-induced enhanced disruption of macroaggregates (>0.25 mm) and large microaggregates (0.05/0.063–0.25 mm). The macro- and large microaggregates can break into primary silt and clay sized particles and small microaggregates, which would in this fractionation scheme fall into MOM fraction smaller than 0.063 mm [32]. According to Chenu and Plante [33], even in the clay-sized fraction many of the particles are in fact microaggregates where OM is encrusted by soil mineral particles. The more effective dispersion with SHMP had less impact on the silt loam soil in comparison to the clay due to higher sand content and lower aggregation in the loam.

**Table 2:** Model coefficients of linear mixed models for fixed effects of treatment (added fiber (FS) and no fiber) and mode of sample dispersion (in sodium hexametaphosphate (SHMP) and in deionized water) for bulk mass fractions and density separated coarse and medium size fractions of clay (Luvic Stagnosol) and silt loam (Dystric Arenosol) soil.

Mass content (g 100 g^−1^ soil)	Clay (Luvic Stagnosol)		Silt loam (Dystric Arenosol)	
	Treatment (FS)	Dispersion (SHMP)	Treatment (FS)	Dispersion (SHMP)
Size fraction 0.25–2 mm	0.097***	−0.094***	0.102***	−0.026
Size fraction 0.063–0.25 mm	0.075***	−0.170***	0.069**	−0.107***
Size fraction <0.063 mm	−0.014***	0.021***	−0.051***	0.063***
POM size fraction 0.25–2 mm	0.312**	−0.110	0.376***	−0.133
MOM size fraction 0.25–2 mm	0.045	−0.072	0.089**	−0.027
POM size fraction 0.063–0.25 mm	0.535***	0.338***	0.726***	−0.450**
MOM size fraction 0.063–0.25 mm	0.021	−0.162***	0.057	−0.060

Asterisks denote p-values of: * < 0.05, ** < 0.01, ***<0.001.

Sodium hexametaphosphate is known as a widely effective dispersant supplying dispersive sodium for replacing binding polyvalent cations and hexametaphosphate anions for complexing actions [[34], [35], [36]]. However, though the difference between the present water and SHMP dispersion treatments was significant, it was relatively small, which indicated that prolonged shaking in water intensified with glass beads provided sufficient mechanical energy to break most of the aggregate structures in the studied soils. The high dispersion efficiency of water was probably contributed by the use of field moist soils instead of air-dried. Upon drying the electrical double-layer surrounding the surfaces becomes thinner enabling stronger inter particle bonding or cementation that can hold even for a one week of complete re-wetting [28].

In soil texture analysis [37], soil aggregate structure is fiercely disrupted by oxidizing organic material with hydrogen peroxide under heating followed by dissolving binding structures in dilute hydrochloric acid. Comparing the textural fractions of particle size analysis (Table 1) to the wet sieved size fractions of C fractionation (Fig. 2, organic matter retained) showed close conformity on both soils. In the textural analysis, the proportions of <0.063 mm and >0.063 mm size fractions were 88 % and 12 % in the clay and 63 % and 37 % in the silt loam, respectively. The corresponding values after the present dispersion in water were 89 % and 11 %, respectively, in the clay and 59 % and 41 %, respectively, in the silt loam soil. With SHMP dispersion, distributions of 90 % and 10 %, respectively, in the clay and 63 % and 37 %, respectively, in the silt loam soil were attained. The congruence in the mass distribution of the size fractions between the different separation methods highlights both the success of the present dispersions and minor difference between the dispersing agents (water and SHMP).

At both sites, the recovered soil mass in the two coarser fractions was increased and that in the fine fraction decreased due to the fiber treatment (Table 2, Fig. 2). These small differences suggest that the fiber amendments appeared in the coarse size range. Furthermore, the observed mass differences in the size fractions between unamended and fiber amended soils were smaller in SHMP dispersed soils in comparison to those dispersed in water, which indicates that the fiber amendment had a strengthening impact on soil aggregates as some aggregates were disrupted with SHMP only. The effect of pulp mill fibers on soil structural stability has also been reported by Rasa et al. [24], who showed reduced risk for soil erosion in clay soil field amended with pulp mill sludge.

### 3.2 Mass proportions of light and heavy material and total C recovery

Consistently with the bulk coarse and medium size fractions, SHMP dispersion tended to show a decreasing effect and fiber addition an increasing effect on the masses of the density separated fractions (Table 2). In exception, an increasing effect of SHMP dispersion was observed in the medium POM of the clay soil. Overall, majority of the soil mass resided in the heavy MOM fractions following density separation (85.4–94.2 % in the clay and 94.6–99.6 % in the silt loam) and the effects of the treatments on the relative mass distributions between POM and MOM were rather small.

The concentration of C in the MOM density fractions was strongly affected by the preceding dispersion treatment such that clearly lower C concentrations were recorded in the heavy fractions of 0.25–2 mm size in both soils and in the heavy fraction of 0.063–0.25 mm size in the clay soil with SHMP dispersion in comparison to water (Table 3). Similar trend was also evident in the <0.063 mm fraction not separated by density though the decrease was significant only in the silt loam. Consequently, on average ca. 4 % and 11 % less of the total C was recovered in the SHMP dispersed samples in comparison to those dispersed in water in the clay (p = 0.14) and silt loam (p = 0.01), respectively. When using water as the dispersion solution, the total recovery of C in the three soil size fractions was 93 ± 1.1 % in the clay soil and 90 ± 1.3 % in the silt loam soil, while with SHMP the corresponding recoveries were 88 ± 1.6 % and 80 ± 2.0 %, respectively. Chan [38] reported SHMP to dissolve organic C resulting in underestimation of the POC fractions. In the present study, any C dissolved during the dispersion phase would be recovered in other fractions and would thus not contribute to loss in total recovery rates. An SHMP-induced increase in dissolving of SOM during the density separation of the coarser fractions would have led to losses of C. However, mass recoveries in the density separation step did not directly support this assumption since total mass recoveries were higher in the SHMP dispersed soils (104 ± 0.4 % in the clay and 98 ± 1.0 % in the silt loam soil) in comparison to the dispersion in water (100 ± 0.4 % in the clay and 94 ± 0.8 % in the silt loam soil). On the other hand, recoveries exceeding 100 % suggest that some SPT tended to remain in the SHMP dispersed samples despite of thorough rinsing, which could mask small losses of C and cause a diluting effect on the C concentrations.

**Table 3:** Characteristics of the POM and MOM fractions of unamended (Control) and wood fiber sludge (FS) amended clay (Luvic Stagnosol) and silt loam (Dystric Arenosol) soil dispersed in water (H_2_O) or in sodium hexametaphosphate (SHMP). The pairwise p-values have been computed based on contrasts from the linear mixed-effects model.

		Clay (Luvic Stagnosol)				Model coeff.		Silt loam (Dystric Arenosol)				Model coeff.	
		Control		FS		Treat. (FS)	Disp. (SHPM)	Control		FS		Treat. (FS)	Disp. (SHPM)
		H_2_O	SHMP	H_2_O	SHMP			H_2_O	SHMP	H_2_O	SHMP		
C (%)	0.25–2 mm POM	30.1a	33.3b	30.9c	35.5d	0.044*	0.120***	31.4a	32.9b	32.4b	36.2c	0.062*	0.079*
	0.25–2 mm MOM	1.7a	1.2b	1.7a	1.2b	0.000	−0.348***	0.4a	0.2b	0.4a	0.2b	0.031	−0.711***
	0.063–0.25 mm POM	29.9a	29.7a	28.7a	28.9a	−0.033	0.001	31.4a	30.0a	31.0a	31.0a	0.018	−0.032
	0.063–0.25 mm MOM	3.6a	2.3b	4.3a	2.1b	0.017	−0.607**	0.4 ab	0.4a	0.5b	0.4 ab	0.095	−0.210
	<0.063 mm MOM	1.9 ab	1.7a	2.2c	2.0bce	0.143*	−0.087	2.4 ab	1.9c	2.6a	2.2bce	0.093	−0.212***
N (%)	0.25–2 mm POM	1.3a	1.5b	1.5b	1.6c	0.088**	0.095**	1.3a	1.4b	1.3a	1.4b	−0.002	0.076**
	0.25–2 mm MOM	0.1 ab	0.1cd	0.1ac	0.1bd	0.004	−0.223*	0.04a	0.04a	0.03a	0.03a	−0.257	0.018
	0.063–0.25 mm POM	1.4a	1.7b	1.5a	1.7b	0.027	0.151***	1.6a	1.5a	1.5a	1.5a	−0.003	−0.015
	0.063–0.25 mm MOM	0.3a	0.2b	0.3a	0.2b	0.008	−0.584**	0.05a	0.06a	0.05a	0.05a	−0.073	0.028
	<0.063 mm MOM	0.2 ab	0.2a	0.2b	0.2 ab	0.149	−0.132	0.2a	0.2b	0.2a	0.2b	0.039	−0.257***
C/N	0.25–2 mm POM	23a	23a	21a	23a	−0.043	0.024	24 ab	24ac	26cd	26bd	0.064*	0.003
	0.25–2 mm MOM	13a	14a	15a	12a	−0.004	−0.125	14 ab	5c	14a	8bce	0.288	−0.728**
	0.063–0.25 mm POM	21a	18b	20c	17d	−0.059*	−0.150***	20a	20a	20a	20a	−0.022	0.045
	0.063–0.25 mm MOM	13a	14a	14a	13a	0.008	−0.022	8a	6b	9c	7a	0.168*	−0.238**
	<0.063 mm MOM	10a	11a	10a	11a	−0.006	0.045	11a	11 ab	11 ab	12b	0.055	0.045

Significance of model coefficients of linear mixed models for fixed effects of treatment are denoted with asterisks (p-values of: * < 0.05, ** < 0.01, ***<0.001), and significant differences between treatments within soil types are denoted with different letters (p < 0.05).

The exact nature of the SHMP-induced interference, clearly more pronounced in the silt loam than in the clay soil, remained unexplained in this study. However, as shaking in deionized water was found sufficient in disrupting soil microaggregates in the field moist soils, and the water treatment also presumably minimized transformations in soil C while maximizing C recovery, further considerations are centered to samples dispersed in water.

### 3.3 Distribution of SOC in size and density fractions

**Fig. 3:**
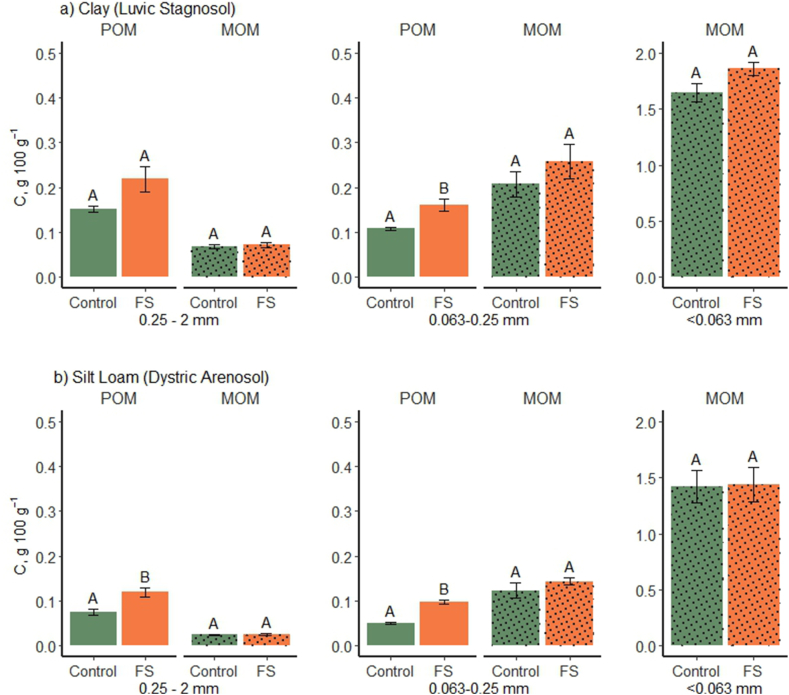
Organic carbon (g 100 g^−1^ soil) in light fractions (POM) and heavy fractions (MOM) in untreated (Control) and woof fiber sludge-amended (FS) soils of a) Clay (Luvic Stagnosol; Jokioinen) and b) Silt Loam (Dystric Arenosol; Maaninka). The values are averages of 4 replicates ± standard error. Significant differences between control and fiber amended soils within soil types and size and density fractions are denoted with different letters (p < 0.05). The pairwise p-values have been computed based on contrasts from the linear mixed-effects model.

In both soils, majority of C (roughly 70–85 % of the total C recovered) resided in the <0.063 mm size fraction assumed to comprise entirely of mineral associated forms (MOC) (Fig. 3). The two coarsest size-fractions 0.063–0.25 mm and 0.25–2 mm contained approximately 15 % and 10 % of the soil C reserves, respectively. The silt and clay fractions tend to constitute the major SOC storage in cultivated croplands, whereas in soils under native perennial vegetation, a considerable proportion of the soil C storage may reside in the coarse fractions as free POM susceptible to degradation when the soil is disturbed [32,39,40]. Differences in the capacity of the particle-size fractions to bind C derives from differences in their specific surface area and mineralogical composition. Active mineral components, namely metal oxides and phyllosilicates, predominating in organo-mineral associations mainly occur in the small size range, whereas coarse particles tend to be composed of minerals with low sorption ability like quartz and plagioclase [[41], [42], [43]].

The two coarser size fractions (0.063–0.25 mm and 0.25–2 mm) were density separated with SPT to isolate the freely occurring light organic carbon (POC) and heavy MOC. Sodium polytungstate allows density gradient separations up to 2.90–3.10 g cm^−3^ [29,44]. In the context of soil organic matter fractionation, densities of 1.6 and 1.8 g cm^−3^ are most used [45]. Increasing the density of the separating solution increases the amount of mineral matter in the floating light fraction while diluting its C concentration. Crow et al. [44] and Cerli et al. [17] reported a density of 1.6 g cm^−3^ to separate maximum amounts of organic material with minimum mineral contamination. At lower densities, organic material ends up in the sinking heavy fraction as the density of soil organic matter tends to range between 1.3 and 1.6 g cm^−3^ [17,46]. As already stated in the Material and methods section above, a more distinct density cut-off was achieved with 1.8 g cm^−3^ than 1.6 g cm^−3^ density with the present soils, which facilitated an easier and more accurate separation.

In the present density fractions, the C concentrations in the light POM fractions were clearly greater than in the heavy MOM (Table 3), which indicates a succeeded exclusion of mineral matter from the POM fractions [11]. The ratios of C to N likewise differed between the POM and MOM fractions such that highest ratios exceeding 20 were found in the coarser POM and ratios around 20 in the finer POM, whereas in the MOM fractions, the C:N ranged roughly between 10 and 15. The SOC density fractions have been shown to differ in average age, decomposition stage, and consequently also in chemical composition such that POM is largely composed of fresh plant residues, whereas MOM is enriched with microbially processed compounds [47,48]. The C to N ratios decrease with degree of decomposition reflecting the compositional changes [49]. Overall, on a global scale, the C to N ratio in the clay-sized fraction is typically around 10, whereas in the bulk soil and coarser size fractions it is on average higher and varies more widely among climate, land use and soil depth [50].

As for the distribution of SOC storage, in the coarsest size fraction (0.25–2 mm), majority of C (70–75 % in the clay and 75–85 % in the silt loam soil) was in the free light fraction and consequently 25–30 % (clay soil) and 15–25 % (silt loam) of C was associated with mineral matter. Going down in particle size to 0.063–0.25 mm, the proportions were roughly turned round as the light POC accounted for 35–40 % and 30–40 % of the C contents in the clay and silt loam soil, respectively, making MOC the dominant form of C in this size fraction (60–65 % in the clay and 60–70 % in the silt loam). This finding was unexpected as the sand-sized particles are hardly thought to contribute to SOC associations as discussed above. It is noticeable that the C concentrations of the density separated MOM fractions in the clay soil were strikingly high and clearly higher when the soils were dispersed in water in comparison to SHMP though the latter method was impaired by reduced total C recovery as discussed above. Despite of this discrepancy and the apparent consistency between obtained particle size distributions in the dispersion treatments and textural analysis, it can be questioned whether the sand-sized MOM originates from stable 0.063–0.25 mm sized aggregate structures that protect OM through mineral associations and by encapsulation of POM [51,52]. Jagadamma and Lal [39] observed enrichment of SOC in the sand sized MOM fraction with increasing bulk soil SOC and suggested that it would be caused by retention of POM heavier than the density used in the study (1.6 g cm^−3^) and thus recovered in the MOM fraction. However, Jindaluang et al. [53] and Yeasmin et al. [54] found SOM associated in heavy mass fractions (representing mainly quartz and feldspar) to be more decomposed than POM and rather resistant to oxidation, but its exact retention mechanism remained unclear.

After density fractionation, the sum of MOM-fractions showed that 88 % and 86 % of the SOC in the unamended clay soil was in heavy mineral associated form when dispersed with water and with SHMP, respectively. In the unamended silt loam soil, the share of MOM of the total SOC was 93 % regardless of the dispersion method. In case merely size separation would have been used after SHMP dispersion in the fractionation as conducted e.g., by Cotrufo et al. [55], shares of SOC considered unprotected (sum of SOC in the >0.063 mm size fractions) would have been 22 % and 14 % in untreated clay and silt loam soil, respectively, while the corresponding values after density separation (sum of POM fractions) were 14 % and 7 %, respectively. Similarly, the review of Gregorich et al. [18] showed size separation to yield higher shares of SOC in the POM fraction of agricultural soils (median value of 19 %) than density separation (median value of 6 %) and they highlighted that fractions separated by size differ in chemical characteristics from those separated by density. Consistently, the higher C:N ratios of the POM fractions in comparison to the MOM in the current study (Table 3) indicate density-dependent quality differences within the size fractions. Overall, though the share of sand-sized MOM of total SOC is small, its nature and stability deserve more attention in future studies to allow valid prediction of the fate of SOC based on fractionation data.

### 3.4 Distribution of fiber-sludge derived SOC in size and density fractions

The fiber amendment was found to increase C in the free POM fractions excluding the coarse POM in the clay soil, whereas in the mineral associated fractions differences between treatments did not turn significant though an increasing trend was apparent in the clay soil (Table 4, Fig. 3). This finding is consistent with the previous perception that the unprotected POM is more sensitive to changes in the management than the stabilized MOM and thus responds stronger to both degradation-promoting changes and increased C inputs [10,38]. A longer time frame and larger C supply might have been needed to induce marked changes in the MOM pools considering the positive signal seen in the clay soil with repeated high C addition. For example, Cavalcante et al. [56] recorded an increase in the silt-sized MOM after 10 years of liquid manure applications. In the study of Baiano and Morra [57], 5-year repeated compost amendment increased SOC in the MOM fraction more than in the coarse size fraction containing POM (an increase of 3.4 g C kg^−1^ soil in MOM vs. 0.3 g C kg^−1^ soil in POM). However, the sandy loam soil used by Baiano and Morra [57] was initially low (ca. 1 %) in SOC. In soils high in SOC or low in clay- and silt-sized mineral particles, the supposed saturation of the mineral sorption capacity may prevent further accumulation of MOM [12,32] as contemplated by Lajtha et al. [58] in relation to a 50-year litter addition treatment showing no SOC accumulation in the MOM fraction on a forested site. In the current study, the clay soil with 78 % of clay- and silt-sized (<0.02 mm) mineral particles would be saturated at MOC content of 3.3 g 100 g^−1^ soil when estimating the sorption capacity according to Hassink et al. [12]. Thus, the measured MOC content of 1.9 g 100 g^−1^ soil in the unamended control soil indicates saturation deficit and therefore potential for MOC accrual on the mineral surfaces. The silt loam soil of Maaninka with 39 % of clay- and silt-sized mineral particles would be saturated at MOC content of 1.8 g 100 g^−1^ soil and the measured MOC content of the control soil was 1.6 g 100 g^−1^ soil which indicates that 87 % of the MOM sorption capacity was already occupied. According to Stewart et al. [59], the SOC accrual is less efficient as the soil approaches saturation and therefore increasing the MOC content in silt loam soil with organic soil amendments is likely more difficult compared to the clay soils with only 57 % of the MOM sorption capacity occupied.

**Table 4:** Model coefficients of linear mixed models for fixed effects of treatment (added fiber (FS) and no fiber) for total carbon content in different size and density fractions of clay (Luvic Stagnosol) and silt loam (Dystric Arenosol) soil.

Carbon content (g 100 g^−1^ soil)	Treatment (FS)	
	Clay (Luvic Stagnosol)	Silt loam (Dystric Arenosol)
POM size fraction 0.25–2 mm	0.348	0.473*
MOM size fraction 0.25–2 mm	0.048	−0.018
POM size fraction 0.063–0.25 mm	0.392*	0.724**
MOM size fraction 0.063–0.25 mm	0.209	0.174
MOM size fraction <0.063 mm	0.124	0.014
Total C in sum of fractions	0.165*	0.043

Asterisks denote p-values of: * < 0.05, ** < 0.01, ***<0.001.

Furthermore, it has been suggested that recalcitrant low-quality amendments i.e., substrates exhibiting low N contents and high C/N ratios and lignin, have less potential in promoting the accumulation of MOM than more labile high-quality substrates that are utilized by microbes more efficiently and thus produce relatively more microbial residues [60]. Therefore, the result indicating that the MOM was not significantly affected after addition of wood fiber sludges with high C/N ratio could be partially attributed to the quality of the used organic soil amendments. However, more recent studies have highlighted the inconsistent effects of litter quality on MOM accumulation [61]. In addition to microbial products, plant-derived components can seemingly contribute directly to MOM and the type of the soil mineral phase likewise plays a role in controlling the formation of MOM [62]. Córdova et al. [63] found greater accumulation of MOM with high- than with low-quality litters but the efficiency of MOM accumulation and the stability of the MOM were higher with the low-quality litters.

Overall, the sum of C in the five individual fractions showed a fiber amendment-induced increase (significant in the clay soil, Table 4) that corresponded to 465 g C m^−2^ (4.65 Mg ha^−1^) in the 10-cm surface layer of the clay and 173 g C m^−2^ (1.73 Mg ha^−1^) in the upper 10 cm of the silt loam soil, when calculating the storage in fiber amended and unamended soil to an equal soil mass using bulk density values of 1.2 g cm^−3^ for the clay soil and 1.3 g cm^−3^ for the silt loam. These increases correspond to approximately 50 % of the amount of C applied within the fiber sludge amendments half to one year before sampling. The clay soil had received an equal sludge application 5 years earlier, of which no significant increase in the soil 0–20 cm surface layer could be detected 4 years later with whole soil total C measurements [24]. The current observation of preservation of the added C agrees with previous studies on paper sludge [27,64], in which a two-phase composition pattern exhibiting a rapid phase followed by slow decay was observed. In their studies, approximately half of the added C was lost during the year following application the decay rate depending on temperature and other soil environment factors. In here, the majority of C added in the fiber sludge being recovered in the POM fractions suggests it to remain vulnerable for mineralization during the coming years. However, in case of saturation deficit on the surfaces of clay and silt-sized particles, the pool of SOC protected by mineral matter may slowly increase with regular additions of organic materials.

## 4 Conclusions

In soil dispersion, which is a crucial step in competent size separation, SHMP showed better ability than deionized water in breaking soil aggregate structures with up to 10–15 % decrease in the mass of 0.25–2 mm and 0.063–0.25 mm size fractions. However, the use of SHMP significantly decreased the total recovery of C, possibly due to analytical interference or inducing C loss through transformations. In the end, deionized water was considered sufficiently efficient in dispersion of field moist soils not consolidated via drying.

The relative distribution of SOC among the five obtained fractions was rather similar between the clay and silt loam soil. Majority, 70–85 %, of the SOC resided in the <0.063 mm fraction, while 5–10 % occurred in the coarse (0.25–2 mm) POM, 1–3% in the coarse MOM, 5 % in the medium (0.063–0.25 mm) POM, and 10 % in the medium MOM. The sand sized particles are hardly expected to protect SOM so the noticeable share of MOM in these fractions, whether bound to stable aggregate structures or truly attached to the coarse particles, deserves to be explored in further studies.

In both the clay and silt loam soil, wood fiber sludge induced an increase in the SOC corresponding to roughly 50 % of the amount of C added within the amendment. The increase was confined to the POC fractions, though in the clay soil having received two batches of the sludge within ca. 5 years, indication of SOC accumulation as MOC could be seen. The POC/POM is known to be less stable than MOC/MOM but nevertheless its accumulation is advantageous considering soil fertility and climate change mitigation.

## Data availability

Data associated with the study is available at https://doi.org/10.5281/zenodo.10377555.

## Ethics declarations

Review and/or approval by an ethics committee was not needed for this study because it contains no human or animal subjects and therefore informed consent was neither applicable.

## Additional information

No additional information is available for this paper.

## CRediT authorship contribution statement

**Riikka Keskinen:** Writing – review & editing, Writing – original draft, Methodology, Investigation, Funding acquisition, Conceptualization. **Johanna Nikama:** Writing – review & editing, Methodology, Investigation, Conceptualization. **Joel Kostensalo:** Writing – review & editing, Writing – original draft, Formal analysis. **Mari Räty:** Writing – review & editing, Resources. **Kimmo Rasa:** Writing – review & editing, Resources. **Helena Soinne:** Writing – review & editing, Writing – original draft, Visualization, Project administration, Methodology, Investigation, Funding acquisition, Conceptualization.

## Declaration of competing interest

The authors declare that they have no known competing financial interests or personal relationships that could have appeared to influence the work reported in this paper.
